# The use of cell phones and radio communication systems to reduce delays in getting help for pregnant women in low- and middle-income countries: a scoping review

**DOI:** 10.3402/gha.v8.28887

**Published:** 2015-09-10

**Authors:** Sunday O. Oyeyemi, Rolf Wynn

**Affiliations:** 1 Accident and Emergency Department, State Specialist Hospital, Akure, Nigeria; 2 Telemedicine and e-Health, Department of Clinical Medicine, Faculty of Health Sciences, UiT The Arctic University of Norway, Tromsø, Norway

**Keywords:** pregnant, childbirth, cell phone, mobile phone, radio communication, mhealth, maternal health services, emergency medical services

## Abstract

**Background:**

Delays in getting medical help are important factors in the deaths of many pregnant women and unborn children in the low- and middle-income countries (LMIC). Studies have suggested that the use of cell phones and radio communication systems might reduce such delays.

**Objectives:**

We review the literature regarding the impact of cell phones and radio communication systems on delays in getting medical help by pregnant women in the LMIC.

**Design:**

Cochrane Library, PubMed, Maternity and Infant care (Ovid), Web of Science (ISI), and Google Scholar were searched for studies relating to the use of cell phones for maternal and child health services, supplemented with hand searches. We included studies in LMIC and in English involving the simple use of cell phones (or radio communication) to either make calls or send text messages.

**Results:**

Fifteen studies met the inclusion criteria. All the studies, while of various designs, demonstrated positive contributory effects of cell phones or radio communication systems in reducing delays experienced by pregnant women in getting medical help.

**Conclusions:**

While the results suggested that cell phones could contribute in reducing delays, more studies of a longer duration are needed to strengthen the finding.

Delays in getting medical help are important factors in the deaths of many pregnant women and unborn children. Delays are a major problem, especially in the countries classified by the World Bank as low- and middle-income countries (LMIC) (1). Many studies have implicated delays as the key factors in the high maternal death rates in sub-Saharan Africa (2, 3). The underlying causes for these delays may be attributed to societal norms, lack of education, long distances to health facilities, weak health systems, and poverty (4). The delays may be classified into three phases: 1) delay in reaching the decision to seek medical help, 2) delay in reaching the health facility, and 3) delay in receiving appropriate and adequate care at the health facility (5).

There are about 5 billion mobile phone subscriptions in the world, of which more than 70% belong to people living in the LMIC (6, 7). We lack data about the mobile phone access of pregnant women specifically, but believe that it would not differ much from the general population of the LMIC. The massive spread of cell phones into the LMIC (7, 8) has been seen as a potential means of leveraging on mobile health (mhealth) to reduce maternal (and infant) death rates. Some projects and pilot programmes using cell phone communication have been initiated in several LMIC to improve maternal and child health. Even though most studies of the use of cell phones in healthcare systems are in the high-income countries (9, 10), the potential impact of mhealth on maternal and child health may be even greater in the LMIC (9). The LMIC struggle with particular health system challenges such as a shortage of health workers and insufficient funding of the national health systems, with a resulting low capacity to meet the health needs of the population (11, 12). Thus, the use of cell phones may have a greater potential for impact in the LMIC, where health services are less accessible or unavailable (9). In certain areas of developing countries, radio communication systems were originally in use before the advent of cell phones and may still be relevant due to lack of cell phone coverage. As far as we know, no reviews have been published that have focused in detail on evidence regarding how cell phones can help in reducing delays in getting help to pregnant women in LMIC. We therefore performed a scoping review to get an overview of the literature (including different types of studies, participants, interventions, outcomes, etc.) documenting the impact of cell phones and radio communication systems on delays in three different phases in getting medical help by pregnant women in the LMIC (13).

## Materials and methods

### Strategy for database search

A literature search limited to the English language was conducted to identify relevant articles in electronic databases. These databases included Cochrane Library, PubMed, Maternity and Infant care (Ovid), Web of Science (ISI), and Google Scholar. The search was performed in June 2015. The same search terms were used in all the databases and these were various combinations of: pregnant women, childbirth, cell phone, mobile phone, radio communication, mobile health, mhealth, maternal health services, emergency medical services, and medical help. The reference lists of the retrieved and relevant articles were also hand searched. The details of the database search can be found in the Supplementary file.

### Inclusion and exclusion of studies

**Fig. 1:**
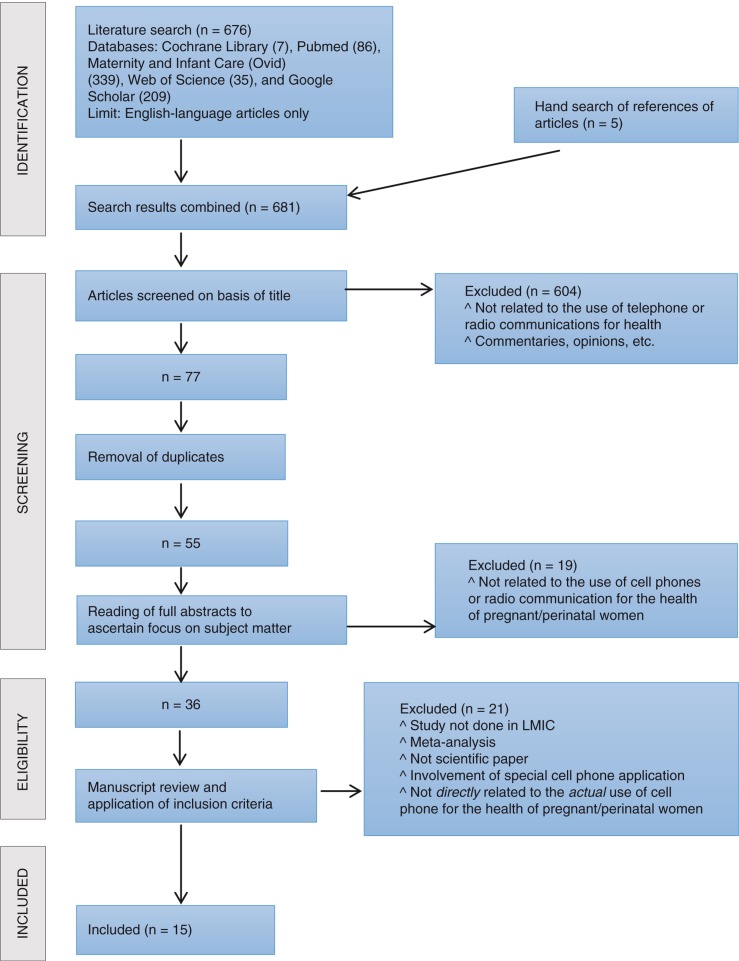
Selection of studies included in the review.

A total of 676 articles were retrieved from the initial electronic search. Hand search of the references of articles yielded an additional five relevant articles. The titles of the articles were screened for relevance, and this drastically reduced the number to 77. This was because many of the articles from Maternity and Infant Care (Ovid) and Google Scholar were either articles with no direct link to the subject matter or to patients; or expert opinions or commentaries on the topic. Duplicates were removed and the number thereby further reduced to 55. The abstracts of the 55 studies were subsequently read to identify studies focusing on the use of cell phones and/or radio communication for maternal and child health, and this narrowed the number of articles to 36. The full text of the 36 studies were read to limit the studies to those relevant to the topic of the review and conducted in LMIC. This yielded the final 15 articles included in this review (see Table 1 and Fig. 1).

**Table 1:** Included articles (*n*=15)

	Articles/authors	Methods; country	Participants	Key findings	Comments
1	Mobile phones as a health communication tool to improve skilled attendance at delivery in Zanzibar: a cluster-randomized controlled trial. – Lund et al., 2012	Cluster randomised controlled trial (primary healthcare facilities as the unit of randomisation); Zanzibar (semi-autonomous part of Tanzania in East Africa)	2,550 pregnant women (1,311 intervention and 1,239 controls)	Significant rise in skilled delivery (in women residing in the urban area)	Possible reduction of Phases I and III delays
2	Satisfaction of healthy pregnant women receiving short message service via mobile phone for prenatal support: a randomized controlled trial. – Jareethum et al., 2008	Randomised controlled trial; Bangkok, Thailand	68 healthy pregnant women	Higher satisfaction and confidence levels with lower anxiety level in the antenatal period in women who received SMS compared with the general antenatal care group	Possible reduction of Phase I delay
3	Mobile phones improve antenatal care attendance in Zanzibar: a cluster randomized controlled trial. – Lund et al., 2014	Cluster randomised controlled trial (primary healthcare facilities as the unit of randomisation); Zanzibar (semi-autonomous part of Tanzania in East Africa)	2,550 pregnant women (1,311 intervention and 1,239 controls)	The intervention markedly increased the proportion of pregnant women attending the prescribed four antenatal care visit Rise in the numbers of preventive health services, as more women with complications were discovered and referred	Reduction of Phases I and III delays
4	Giving cell phones to pregnant women and improving services may increase primary health facility utilization: a case-control study of a Nigerian project. – Oyeyemi and Wynn, 2014	Case-control study; Nigeria	3,230 women who registered before birth of which 1,280 gave birth at health facilities	Giving cell phones to pregnant women may increase primary healthcare system utilisation No difference in odds of occurrence of the five major causes of maternal deaths between the intervention area and the control area (OR = 1)	Reduction of Phases I, II and III delays
5	A pilot study using interactive SMS support groups to prevent mother-to-child HIV transmission in South Africa. – Dean et al., 2012	Mixed design; South Africa	7 HIV-positive pregnant women	The challenges of stigma and logistics were comparatively overcome while the participants disclosed overall satisfaction	Reduction of Phase I delays
6	Impact of mobile telephone on maternal health service care: a case of Njoro Division. – Fedha, 2014	Prospective randomly controlled study; Kenya	397 pregnant women (191 as study group and 206 as control group)	Significantly more women in the study group had more than four antenatal visits (p=0.002). The proportion of women who delivered in hospital was significantly higher in the study group (88% vs. 72.8%, p<0.001).	Reduction of Phases II and III delays
7	Facilitating emergency obstetric care through transportation and communication, Bo, Sierra Leone. – Samai and Sengeh, 1997	Interventional; Sierra Leone	Number of participants not specified	Radio communication aided transport can help more women with complications reach hospital, and may improve their chances of survival	Reduction of Phase II delay
8	Some information and communication technologies and their effect on maternal health in rural Uganda. A summary of research findings prepared for the African Development Forum. – Musoke, 1999	Interventional; Uganda	Number of participants not specified	The intervention, Rural Extended Services and Care for Ultimate Emergency Relief (RESCUER) increased the number of deliveries under trained personnel and increased referrals to health units	Reduction of Phase II and III delays
9	The Aceh Besar midwives with mobile phones project: design and evaluation perspectives using the information and communication technologies for healthcare development model. – Chib, 2010	Quantitative and qualitative research methods; Indonesia	121 midwives randomly allocated to mobile phone group, 102 to control group	Mobile phones enhanced access to information and reduced response time to difficult cases Enhancement of communication networks among the health workers, and also with the community More efficient data collection Strengthened referral system	Reduction of Phase III delays
10	Working with midwives to improve maternal health in rural Ghana. – Matthews and Walley, 2004	Interventional; Ghana	Number of participants not specified	Rural midwives in the project felt more supported as care givers in isolated areas Maternal and perinatal mortality rates were still high despite intervention The most effective factor in reducing maternal mortality may be an informed community	Possible reduction of Phase III delays
11	Does the upgrading of the radio communications network in health facilities reduce the delay in the referral of obstetric emergencies in Southern Malawi? – Lungu and Ratsma, 2007	Interventional; Malawi	Number of participants not specified	The intervention (radio network system) significantly improved the time interval between the decision to refer and arrival of transport. Although, most transportation still took too long when considering cases such as postpartum haemorrhage	Reduction of Phase II delay
12	Antenatal health promotion via short message service at a midwife obstetrics unit in South Africa: a mixed methods study. – Lau et al., 2014	Mixed method; South Africa	206 pregnant women (intervention group: 102 received text messages while the control group: 104 received no text messages)	The SMS was a wanted reminder and a source of positive motivation for the pregnant women It was seen as extended care from the health facility The intervention fell short of improving antenatal health knowledge	Possible reduction of Phase I delay
13	Designing and Implementing an Innovative SMS-based alert system (RapidSMS-MCH) to monitor pregnancy and reduce maternal and child deaths in Rwanda. – Ngabo et al., 2012	Interventional; Rwanda	A total of 11,502 pregnancies were monitored	A 27% increase in the number of women reaching the facility to deliver (facility-based deliveries) following the intervention. Delay was reduced through SMS sent to alert the health system for timely and appropriate medical intervention	Reduction of Phase I, II, and III delays
14	Toll-free mobile communication: overcoming barriers in maternal and neonatal emergencies in rural Bangladesh. – Huq et al., 2014	Qualitative research method (through in-depth interviews, semi-structured interviews, and focus group discussion); Bangladesh	12 Community Skilled Birth Attendants (CSBA) and 14 mothers with their husbands’ prior to intervention; At intervention end, 6 CSBA for in-depth interview; Semi-structured interviews for all 27 CSBA engaged in the intervention; and one focus group discussion with 10 mothers who recently delivered	Community skilled birth attendants made prompt visits to the pregnant women following reports of ‘complications’ through mobile phones The use of toll-free mobile phones led to the provision of more rapid services to pregnant women	Reduction of Phase III delay
15	Midwives’ cell phone use and health knowledge in rural communities. – Lee, Chib and Kim, 2011	Survey/quantitative; Indonesia	223 village midwives	Cell phone use was positively associated with access to institutional resources Access to institutional resources had a direct positive effect on midwives’ health knowledge Midwives’ health knowledge was found to correlate positively with their self-efficacy	Reduction of Phase III delay

### Inclusion and exclusion criteria

All studies carried out in the LMIC involving the simple use of cell phones or radio communication to either make calls or send text messages/short message services (SMS) were considered for inclusion, while all studies from the high-income countries were excluded. We used the World Bank's classification to identify the countries belonging to the LMIC category (1). Studies involving the use of e-mails, videos, the Internet, any special phone applications, or the downloading of data by the participants, were excluded.

### Data synthesis and analysis

The 15 final studies were categorised into different groups according to the phases of delay addressed in each study. The key findings in each article in the various categories were abridged and the common findings in the various groups identified. However, while some studies addressed one phase of delay, many addressed multiple phases.

## Results

A total of 681 articles were retrieved, of which 15 met the inclusion criteria. The studies had various theoretical bases and designs. Four of these studies were randomised controlled trials; two were from the same project in Zanzibar, Tanzania (14, 15), one from Kenya (16), and one from Thailand (17). One was a case-control study from Nigeria (18). Three had mixed designs; they were from South Africa (19, 20) and Indonesia (21). Five had an interventional design format, and were from Rwanda (22), Ghana (23), Malawi (24), Sierra Leone (25), and Uganda (26). One was a qualitative study from Bangladesh (27) and the last one was a survey from Indonesia (28). The studies were grouped into three categories according to the phase(s) of delay addressed (or considered to have been addressed) in them. Some studies were found to belong to two or three categories (see Table 1).

### Phase I delay: delay in reaching the decision to seek medical help

Seven of the studies (14, 15, 17–20, 22) treated the topic of delay in reaching the decision to seek medical help (i.e. Phase I delay). The interventions in the studies include sending information through SMS to the pregnant women – unidirectionally (14, 17, 20, 22), and sending SMS from the pregnant women to the health facilities – bidirectionally (19), and/or making calls to the health facilities (and vice versa) (14, 15, 18).

#### Outcomes of interventions

None of the studies quantified the effects of cell phones use on Phase I delays; however, Dean et al. (19) reported that the use of cell phones helped to overcome stigma and logistic challenges in HIV-positive pregnant women in getting medical care. Jareethum et al. (17) found that the women who received text messages had significantly higher satisfaction levels and confidence levels and significantly lower anxiety levels than the control group before birth and during labour. However, Lau et al. (20) found that an SMS intervention did not improve antenatal health knowledge, as no statistically significant difference was seen between the intervention and control groups, although substantial loss to follow-up (43%) was recorded during the study. Nevertheless, the authors found that responses from the focus group showed that the use of SMS was seen as a welcome reminder and source of motivation, and also as a form of extended care from the health facility.

### Phase II delay: delay in reaching the health facility

Six of the studies (16, 18, 22, 24–26) treated the topic of delay in reaching the health facility (i.e. Phase II delay). The interventions in these studies include the provision of forms of transportation supported by cell phones (18, 22), or radio communication and telephone systems (16, 24–26).

#### Outcomes of interventions

In the study by Lungu and Ratsma (24), the focus was on the period between getting the pregnant woman from the district health office to the hospital. The median time interval between the decision to refer and the arrival of the vehicle was 3 h in the pre-intervention period and 2 h and 3 min in the post-intervention period (*p*<0.02). There were increases in both the number of admissions and the number of emergency obstetric cases referred to the hospital in the post-intervention period. Samai and Sengeh (25) reported that the radio communication system intervention eliminated the time spent by the community health officer to travel by motorbike from the farthest peripheral health units to the referral hospital. This represents about 2 h removed from the Phase II delay. Consequently, the obstetric case fatality rate in the project area declined from 20% to 10% in the post-intervention period.

Fedha (16) reported that 88% of those exposed to mhealth through telephone prompts and advice about their health gave birth in a hospital as compared to 72.8% of those who were not exposed to mhealth (*p*<0.001). Oyeyemi and Wynn (18), reported significantly higher primary healthcare facility utilisation in the area where cell phones were distributed to women attending antenatal care when compared to the area where cell phones were not distributed (*p*<0.001). Ngabo et al. (22) reported that the rates of facility-based deliveries varied from a minimum rate before the intervention of 72% (in January 2010) to a maximum rate of 92% following the intervention (in March 2011). However, few details and no further statistics regarding the rates were given, which make it difficult to assess the impact of the intervention itself on the rates.

### Phase III delay: delay in receiving adequate and appropriate care at the facility

Ten studies (14–16, 18, 21–23, 26–28) addressed delays in receiving adequate and appropriate care at the health facility, that is, Phase III delay.

#### Outcomes of interventions

Lund et al. (14) reported an increase in skilled attendance at childbirth as 60% of the pregnant women in the intervention group got skilled attendance while this was the case with 47% in the control group (OR 5.73; 95% CI 1.51–21.81). However, this was seen only in the urban women and not in the rural women. Lund et al. (15) found that the use of cell phones by the pregnant women was associated with an increase in antenatal care attendance, and 44% of the women exposed to mhealth received four or more antenatal care visits compared to 31% of those who were not exposed to mhealth (OR 2.39; 95% CI 1.03–5.55). They further reported improved time management and quality of antenatal care services in the intervention group, although this finding was not statistically significant. In the intervention group, more antepartum complications were referred, suggesting that a higher proportion of the women with pregnancy-related complications was identified and subsequently treated in this group than in the control group. Fedha (16) reported a reduction in missed appointments in pregnant women using cell phones as only 3.6% of those exposed to mhealth had less than four antenatal visits while this was the case with 9.7% of those not exposed to mhealth (*p*=0.002). The summary account of the Rural Extended Services and Care for Ultimate Emergency Relief (RESCUER) project given by Musoke (26) reported that the problems of professional isolation of the rural health workers, panicky and uncertain management of complications, and delayed referrals were solved by the project. Chib (21) found that mobile phones appeared to increase the confidence of the midwife study group in solving difficult problems. Lee et al. (28) found that midwives’ cell phone use was positively associated with access to institutional (*p*<0.01) and peer (*p*<0.05) resources. Their results further showed that access to institutional resources had a direct positive effect on midwives’ health knowledge (*p*<0.001), although access to peer resources did not. Midwives’ health knowledge was found to correlate positively with midwives’ efficacy (*p*<0.05). Huq et al. (27) found that the use of toll-free mobile phones led to the provision of more rapid services to pregnant women. Ngabo et al. (22) found that delay was reduced through SMS sent to alert the health system for timely and appropriate medical intervention. However, Oyeyemi and Wynn (18) found that there was no difference in the odds of occurrence of the five major causes of maternal deaths between the intervention area and the control area (OR=1).

## Discussion

Cell phones are becoming pervasive, and even poorer people in the LMIC often have access to this technology (6, 7, 18, 29). While smart phones with Internet access are also becoming increasingly common in many places (8, 30, 31), they are more expensive and not within the reach of all.

As health services in the LMIC are making attempts at taking advantage of cell phones in their efforts to help those in need, relatively few high-quality studies have been conducted in the LMIC evaluating these efforts (32, 33). This review is, to our knowledge, the first that has focused in detail on evidence regarding how the simple use of cell phones can help in reducing delays in getting help to pregnant women in LMIC.

All of the studies used in this review demonstrated positive contributory effects of cell phones or radio communication systems in reducing phases of delay experienced by pregnant women in getting medical help in the LMIC. However, as the design and the study outcomes of the studies varied a lot, it was difficult to compare them directly to each other or aggregate the findings. The effects were demonstrated in various ways by the studies such as: increased facility utilisation, increased antenatal care attendance, reduced missed hospital appointments, increased facility-based deliveries, higher confidence levels, and lower anxiety levels. The total time spent on transporting a pregnant woman to the medical facility, where drivers or vehicles were equipped with means of communication (Phase II delay), was also shown to be reduced markedly. The use of cell phones was also found to reduce the delay experienced in receiving timely and appropriate care at the facilities. This may have been achieved through better time management and capacity improvement. Overall, these studies showed that utilising cell phones might reduce all the three phases of delay experienced by pregnant women in the LMIC.

However, while the studies have suggested the usefulness of cell phones and radio communication in reducing delays, none of the studies we have reviewed have dwelt on the cost effectiveness and long-term sustainability of the use of the technology (34). This is probably because most of the included studies were based on pilot projects. However, some of these projects might be continued and later result in more extensive studies. There was a clear variation in the quality of the studies, and different theoretical bases and designs had been utilised, with only a handful of RCTs identified (35). While we consider the findings of the review promising, we believe there is a need for more extensive studies of longer duration to determine effects over sustained periods of time. While this may not be relatively easy to carry out in resource-poor settings, we believe more studies with an RCT design and other high-quality designs will help increase the quality of the evidence.

It is a potential weakness of the study that only one researcher collected the data, as this might entail a risk of rejecting relevant reports (36). Furthermore, while we believe the search performed in the study was relatively extensive, we cannot rule out that using other search terms or terms with different wordings, wild cards, and so on, could have resulted in other relevant studies being identified. The number of studies included in this review is relatively small as the number of studies in this area is still rather limited. While we have been able to capture studies from various LMIC, we might have missed some studies that have been published in languages other than English.

## Conclusions

The literature available suggests that cell phones can contribute in reducing the various phases of delays in getting help to pregnant women in LMIC. However, there are relatively few studies on this topic, and they have various designs and study outcome measures. There is a need for more RCTs and other high-quality studies that examine potential benefits over longer periods of time.

## Supplementary Material

The use of cell phones and radio communication systems to reduce delays in getting help for pregnant women in low- and middle-income countries: a scoping review
