# Supplementary material for: The use of cell phones and radio communication systems to reduce delays in getting help for pregnant women in low- and middle-income countries: a scoping review
Source: Glob Health Action. 2015 Sep 10;8:10.3402/gha.v8.28887. doi: 10.3402/gha.v8.28887 (PMC4567587; doi:10.3402/gha.v8.28887)
Supplement: The use of cell phones and radio communication systems to reduce delays in getting help for pregnant women in low- and middle-income countries: a scoping review [file GHA-8-28887-s001.pdf]

Supplemental material to the article 'The use of cell phones and radio communication systems to reduce delays in getting help for pregnant women in low and middle income countries: A scoping review'

Details of the search terms

1. Cochrane Library

Date Run: 15/06/15

Description:

ID	Search	Hits
#1	MeSH descriptor: [(Pregnant Women]) explode all trees	100
#2	MeSH descriptor: [(Parturition]) explode all trees	257
#3	pregnant next wom*:ti,ab,kw (Word variations have been searched)	5031
#4	"childbirth":ti,ab,kw (Word variations have been searched)	1322
#5	MeSH descriptor: [(Cell Phones]) explode all trees	454
#6	cell, mobile next phone:ti,ab,kw (Word variations have been searched)	242
#7	radio next communication:ti,ab,kw (Word variations have been searched)	3
#8	MeSH descriptor: [(Maternal Health Services]) explode all trees	1651
#9	MeSH descriptor: [(Emergency Medical Services]) explode all trees	3049
#10	medical next help:ti,ab,kw (Word variations have been searched)	33
#11	#1 or #2 or #3 or #4	6125
#12	#5 or #6 or #7	506
#13	#8 or #9 or #10	4723
#14	#11 and #12 and #13	7

2. Pubmed

Date Run: 15/06/15

Search strategy: ((((((pregnant women[(MeSH Terms])) OR childbirth[(MeSH Terms])) OR pregnant women) OR childbirth)) AND ((((((((cell phones[(MeSH Terms])) OR mobile phones[(MeSH Terms])) OR mobile health[(MeSH Terms])) OR cell phones) OR mobile phones) OR mobile health) OR mhealth) OR radio communication)) AND (((((maternal health services[(MeSH Terms])) OR emergency medical services[(MeSH Terms])) OR maternal health services) OR emergency medical services) OR medical help)

Search hits = 86

3. Maternity and Infant Care (Ovid)

Date Run: 15/06/15

Search strategy: (((((pregnant wom* or childbirth) and cell phone*) or mobile phone* or radio communication) and maternal health service*) or emergency medical service* or medical help).af.

Number of hits = 339

4. Web of Science (ISI)

Date Run: 15/06/15

Search strategy: TOPIC: (pregnant wom* OR childbirth) *AND* TOPIC: (cell* phone* OR mobile phone* OR radio communication) *AND* TOPIC: (maternal health service* OR emergency medical service* OR medical help)

Timespan: All years.

Search language=English

Number of hits = 35

5. Google Scholar

Date Run: 15/06/15

Search strategy: ("pregnant wom*" OR childbirth) AND ("cell* phone*" OR "mobile phone*") AND ("maternal health service*" OR "emergency medical service*" OR "medical help")

Number of hits = 209
